# Effects of Electrodes Layout and Filler Scale on Percolation Threshold and Piezoresistivity Performances of a Cementitious-Based Geocomposite

**DOI:** 10.3390/nano12101734

**Published:** 2022-05-19

**Authors:** Mohammadmahdi Abedi, Raul Fangueiro, António Gomes Correia

**Affiliations:** 1 Department of Civil Engineering, ISISE, University of Minho, Campus de Azurém, 4800-058 Guimarães, Portugal; mohammadmehdi.abedi@gmail.com (M.A.); agc@civil.uminho.pt (A.G.C.); 2 Department of Mechanical Engineering, University of Minho, Campus de Azurém, 4800-058 Guimarães, Portugal; 3 Centre for Textile Science and Technology, School of Engineering, University of Minho, 4800-058 Guimarães, Portugal

**Keywords:** self-sensing, stabilised sand, matrix shape, matrix scale, filler scale, percolation threshold

## Abstract

An extensive experimental study was conducted to investigate the co-effects of surface area and distance between electrodes as well as filler scales on the percolation threshold of piezoresistive cement-stabilised sand. In this route, the electrical resistivity of numerous specimens of different sizes and composed of different content of carbon-based conductive fillers was measured, including carbon nanotubes (CNTs), graphene nanoplatelets (GNPs), and carbon fibres (CFs) with different aspect ratios. In addition, the numerical relations between the electrical percolation threshold and matrix dimensions were expressed for different conductive fillers. Furthermore, the electrical percolation threshold of two large-scale specimens with different shapes (a 10 × 10 × 85 cm^3^ beam, and a 15 cm size cube) were predicted through numerical relations, and their piezoresistivity performances were investigated under compression cyclic loading (cube) and flexural cyclic loading (beam). The mechanical properties of the specimens were also evaluated. The results showed that the changes in the length, width, and thickness of the matrix surrounded between electrodes had a significant effect on the electrical percolation threshold. However, the effects of length changes on the percolation threshold were greater than the width and thickness changes. Generally, increasing the aspect ratio of the conductive fillers caused a reduction in the electrical percolation threshold of the cementitious geocomposite. The appropriate piezoresistivity response of the large-scale specimens composed of filler content equal to their percolation threshold (obtained by the numerical relation presented in this study) showed the adequacy of the results in terms of threshold dosage prediction and self-sensing geocomposite design. The results of this study addressed a crucial factor for the design of self-sensing composites and pave the way for the development of field-applicable, smart, cementitious geocomposite.

## 1. Introduction

**Figure 1:**
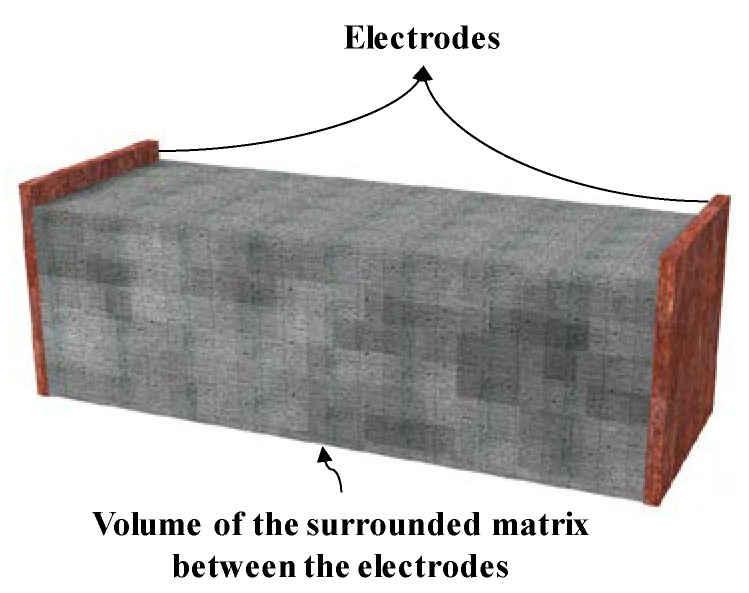
The volume of the surrounded matrix between the electrodes.

Intrinsic, self-sensing, cementitious-based geocomposites (SCGs) are ideal engineering materials for infrastructure-monitoring applications. These intelligent materials can help to develop smart infrastructure integrated with health monitoring and sensing abilities, thus improving the safety, serviceability, durability, and reliability of the infrastructure [1,2]. SCGs have provided a new approach for maintaining sustainable development in roller-compacted concrete dams, rammed earth, ground improvement, and particularly in structural layers in transportation infrastructures, especially in critical zones, such as transition zones [3,4]. A piezoresistive cementitious-based geocomposite is a compounded material, which is composed of a conductive phase distributed in a non-conductive matrix. The conductive phase is often made with one carbon nanomaterial or a mixture of carbon nanomaterials (CNMs) or metallic alloys with different geometrical shapes [5,6,7,8], whereas the non-conductive cementitious phase, or the matrix of the sensor, is commonly compacted cemented sand, mortar, and concrete [9,10,11]. Indeed, the conductive phase is vital for providing the piezoresistive composites with the ability to sense deflection, strain, stress, cracks, temperature, and humidity [12]. The conductive phase dopes the non-conductive phase through two primary mechanisms, namely, percolation and quantum tunnelling effect [13,14]. The percolation phenomenon can be explained by the formation of randomly conductive paths that contribute to increased electrical conductivity [15]. However, studies have indicated that the percolation mechanism cannot justify and interpret the piezoresistivity of sensors containing discontinuous conductive fillers. Hence, to explain the electron movement in the material, quantum tunnelling effect theory is also often required for the exploration of electrical conductivity in the quantum realm [16]. Based on this theory, there is also a non-zero probability that electrons with lower energy can overcome a potential energy barrier, and thus, even with high electrical resistivity and a low external voltage, a minority of electrons can still create a current and move to a place with low potential energy [17,18,19]. The distance beyond which the electrons are not able to pass through a non-conductive matrix or at which tunnelling conductivity becomes invalid is called the “tunnelling distance.” Considering both the percolation mechanism and quantum tunnelling effect, the critical volume fraction of a conductive phase at which a composite can undergo a transition from a non-conductor to a conductor is called the “percolation threshold” [15,20]. However, percolation does not imply a fully physical connection, owing to the tunnelling conductivity. Indeed, percolation threshold content and type of the filler are the most important factors to achieve economical self-sensing cementitious composites with proper mechanical properties and potential for use in the field [3,6,21]. Literature assessment shows that many studies have been conducted on the factors affecting the percolation threshold including the type of conductive fillers, their aspect ratio and geometrical shapes, dispersion, type of non-conductive matrix, size and shape of aggregate, etc. [22,23,24,25,26]. However, to transfer laboratory findings to field applications, it is crucial to study the effects of different measurement systems and electrode configurations on the percolation threshold. In field applications, many laboratory measurement methods may lose their effectiveness due to limitations and scale changes. In addition, in field applications, particularly in bulk forms, by changing the distance, shape and cross-section of the electrodes, the percolation threshold might be changed due to factors such as polarization and contact resistance between electrodes and composite [2]. Chacko et al. [27] and Banthia et al. [28] assessed the effect of electrode distance on the electrical resistivity of cementitious composites. They showed that the measured electrical resistivity increased with rising electrode space. Indeed, they reported that the electrical resistivity would become constant when the space of electrodes reached a threshold. Based on the hypothesis proposed by Banthia et al. [15,28], the diminished capacitance effect with rising electrode spaces may be the reason for this phenomenon. Furthermore, a larger space of electrodes may also reduce interference from the surface resistance while measuring volume resistance [15]. Although many studies have assessed the effects of electrodes configuration on the electrical resistance of cementitious composites, the coeffects of the electrode surface area and electrode distance on the percolation threshold have received less attention. The importance of this issue becomes more apparent when a special electrode layout is required in field applications, due to special constraints such as size or physical shape. Indeed, insufficient addressing of this point has led to few successful experiences in self-sensing composites employment in the field, particularly in bulk form. The co-effects of electrode distance and surface area, which have great effects on the percolation threshold content, can be reflected in the boundary conditions or volume of the surrounded matrix between the electrodes (Figure 1). Accordingly, in this study, the volume of the matrix refers to the volume surrounded between the two electrodes which means considering the combined effects of electrode surface area and electrode space. This approach has a crucial contribution to the design for the field application of self-sensing composites.

Hence, in order to address this issue, different content of carbon-based conductive fillers was dispersed into the specimens fabricated using compacted cementitious stabilised sand of different sizes and shapes. The copper electrodes were installed on both ends of the specimens. In this investigation, carbon microfibres (CFs) and carbon nanotubes (CNTs) with different lengths and a hybrid combination of CNT and graphene nanoplatelets (GNPs) were used as conductive fillers. In each case, the electrical resistance of the specimens was measured after 28 days of hydration in order to achieve the percolation threshold of the specimens. The numerical relations between the percolation threshold, volumetric electrical resistance, and matrix volume were also expressed for different conductive fillers. Finally, two large-scale specimens were prepared using the obtained numerical relations to ensure their adequacy. The mechanical performance and piezoresistivity behaviour of these specimens were also investigated under compression and flexural loading cycles. Indeed, the present study elucidates the path for passing from small-scale laboratory investigations towards large-scale self-sensing geocomposites.

## 2. Materials and Methods

### 2.1. Materials

In order to evaluate the effects of the aspect ratio of the fillers on the electrical percolation threshold, three types of multiwall carbon nanotubes (MWCNTs) and CFs with different lengths were used. The GNPs used in this study were of a multilayer variety with a diameter of approximately 5–10 µm. Carbon nanomaterials and CFs were supplied by Sigma Aldrich (Lisbon, Portugal) and Toho Tenax (Tokyo, Japan), respectively. Additional specifications of these carbon-based fillers are presented in Table 1 [6,29,30].

A compatible non-covalent surfactant with a central hydrophobic chain of polyoxypropylene (PPO) and two hydrophilic side chains of polyoxyethylene (PEO), Pluronic F-127 (Sigma Aldrich), was used to disperse the CNMs. In addition, tributyl phosphate (TBP, 97%, Sigma Aldrich) was used as an antifoaming agent and dispersion booster, according to a previous study [29]. In this study, siliceous and clean particles of CEN-standard sand were used. The classification of this sand following the unified soil classification system (USC) was a well-graded class. The physical properties of the sand are listed in Table 2. The grading was measured by sieving according to ISO 679: 2009 and EN 196-1 standard requirements. In this study, ordinary Portland cement type I (CEM I 42.5R) from Secil (Lisbon, Portugal) was also used as a binder for SCG fabrication [30].

### 2.2. Samples Preparation

**Figure 2:**
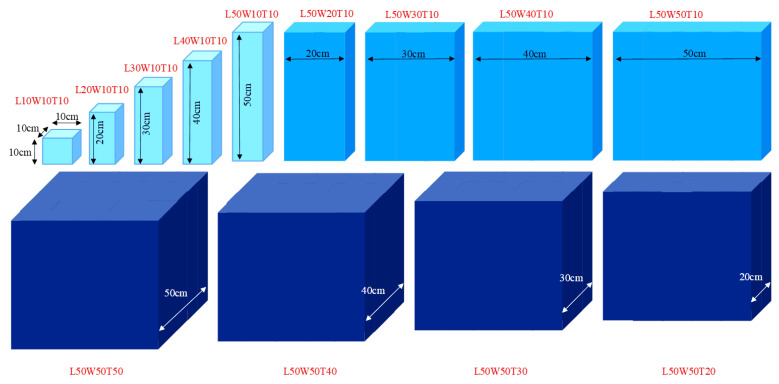
Schematic diagram of the specimens’ dimensions (reinforced by hybrid CNT/GNP, 1:1), L: length, W: width, T: thickness.

The benefits of the synergistic effects of CNT/GNPs when combining them to develop economical self-sensing composites with high sensitivity and low percolation thresholds have been mentioned in several studies [6,31,32,33,34,35,36,37]. Based on this, different contents of CNT/GNP combination (at a 1:1 ratio) were used to investigate the role of the matrix shape and size effects. The specimens were fabricated by gradually increasing the dimensions in different directions, as shown in Figure 2.

**Figure 3:**
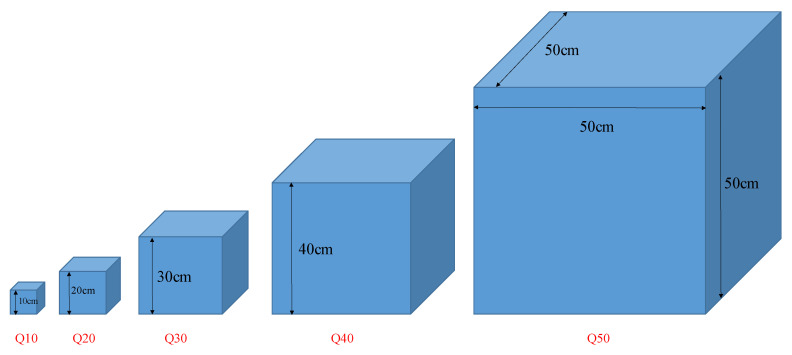
Schematic diagram of the specimens’ dimensions (reinforced by different length of CF and CNT, separately).

In order to evaluate the effects of the aspect ratio of the conductive fillers on the electrical percolation threshold in different matrix volumes (surrounded between electrodes), three different lengths of CF and CNT at different content were separately incorporated into cubic specimens with similar aspect ratios. The dimensions of the specimens gradually increased, as shown in Figure 3. The reason for choosing cubic specimens was to ignore the effect of changes in the aspect ratio of the matrix.

Since, in sand stabilisation, the cement content usually varies by approximately 10% owing to the target strength of the sand–cement [38,39], in this study, 10% cement (by the weight of the dry sand) was also utilized for SCG preparation. Based on the literature [3,21,40], the optimum water content for the CNM-reinforced stabilised sand with similar cement content and sand grading was around 7%. Hence, the amount of water content for all the specimens was considered to be 7% (by the weight of the dry sand) and they were fabricated by the compaction methods. In this route, the CNMs were first dispersed into water through a compatible and effective dispersion method [29,30]. Then, cement and sand were added to a steel bowl (Grade 304) and blended with a stainless-steel (Grade 304) blade at a rotational speed of 140 rpm for 3 min. Then, the CNM suspensions were sprayed into the mixture and blended at a speed of 285 rpm for another 3 min. In the case of CF, the fibre was first de-bundled through the air in a specialized flask at high pressure and blended at a speed of 285 rpm with sand and cement for 5 min. Then, water was sprayed into the mixture and blended at the same speed for another 3 min. Thereafter, the mixture was stored into a plastic bag to retain a constant moisture content. The split moulds were filled with the wet mixture in three equal-height layers. For each layer, the well-mixed wet mixture was poured into the split mould and then compacted carefully using a metallic electrical hammer to the desired height (controlled by Vernier calliper of accuracy 0.02 mm). The compaction of each layer continued as long as the weight of the specimen was not changed (to an accuracy of 0.5 g). In all specimens, two copper meshes (mesh size ≈ 2.5 mm × 2.5 mm) with dimensions equal to the cross-section of the specimen were placed at both ends as electrodes (along the length, top, and bottom in Figure 2 and Figure 3) to completely cover the matrix. For the piezoresistivity behaviour of the geocomposite only one specimen was used in each case.

### 2.3. Electrical Resistivity Measurement

As mentioned previously, in this study, the volume of the matrix refers to the volume of the cross-section between the two electrodes (Figure 1). For this reason, in each case, the size of the electrode was considered equal to the cross-sectional size of the specimens and placed at the boundary of them to completely surround the specimen.

The biphasic DC electrical measurement approach was used in this study to avoid the polarization effects [41]. The biphasic DC method is an effective technique to determine fractional changes in the electrical resistance of cementitious composites [41,42].

To assess the electrical resistance of the specimens, a periodic measure/discharge cycle in the form of a 6 Hz square wave ranging from −5 V to +5 V with a duty cycle of 50% was used. The periodic signal was supplied by National Instruments, equipped with the Arduino Mega 2560 R3 microcontroller board (Pin 10 and 5).

**Figure 4:**
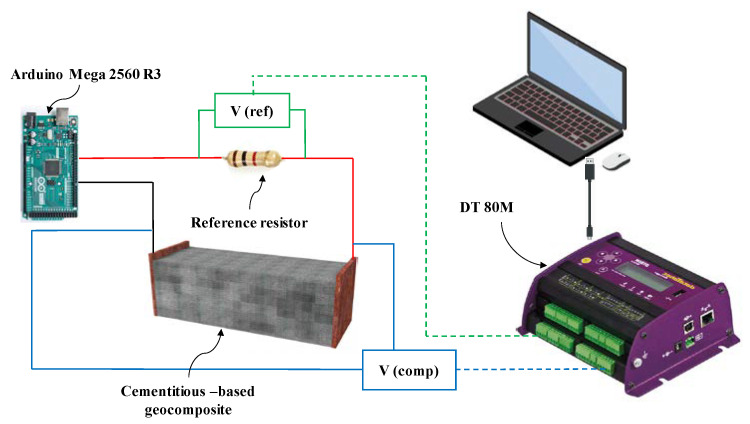
Experimental test configuration for electrical resistance measurement.

In this way, the specimens were oven-dried at 70 °C for 72 h to avoid the effects of moisture content on the electrical conductivity values and were then connected in series to a 1000 Ω reference resistor as shown in Figure 4.

The potential differences between *V_(ref)_* and *V_(comp)_* were measured using a data taker DT 80M (accuracy: 10^−5^ V) at time instants of 80% of the positive constant part of the biphasic signal [41]. Equation (1) was used to calculate the electric current (I) flowing in this circuit, where *V*_(*ref*)_ is the voltage drop across the reference resistor and *R*_(*ref*)_ is the electrical resistance of this resistor (1000 Ω). Due to the complete polarization of the composite, the electrical current (I) achieved a plateau, and the electrical resistance *R*_(*comp*)_ was calculated using Equation (2) [41,42].

(1) $$I=\frac{V_{\left(ref\right)}}{R_{\left(ref\right)}}$$

(2) $$R_{\left(comp\right)}=\frac{V_{\left(comp\right)}}{I}$$

The volumetric electrical resistance (*ρ*) for the system composite and electrodes can be estimated using the electrical resistance *R*_(*comp*)_, as shown in Equation (3). It is vital to remember that this value represents more than just the specimen genuine electrical resistivity. The electrical resistance of the electrodes and the contact resistance between composite and electrodes are two other smaller electrical resistances that are related to this characteristic.

(3) $$\rho =\frac{R_{\left(comp\right)}}{L}\times A$$

where *A* is the contact surface area between the electrode and the specimen (cross-section), and *L* is the spacing between the electrodes (length of the specimen). The results were plotted as the volumetric electrical resistance vs. conductive filler content diagrams [15,43,44,45].

The percolation threshold content was also calculated from the derivative (dy/dx = 0) of the relationship between the conductive filler content and the volumetric electrical resistance of the specimens.

### 2.4. Piezoresistivity Investigations

**Figure 5:**
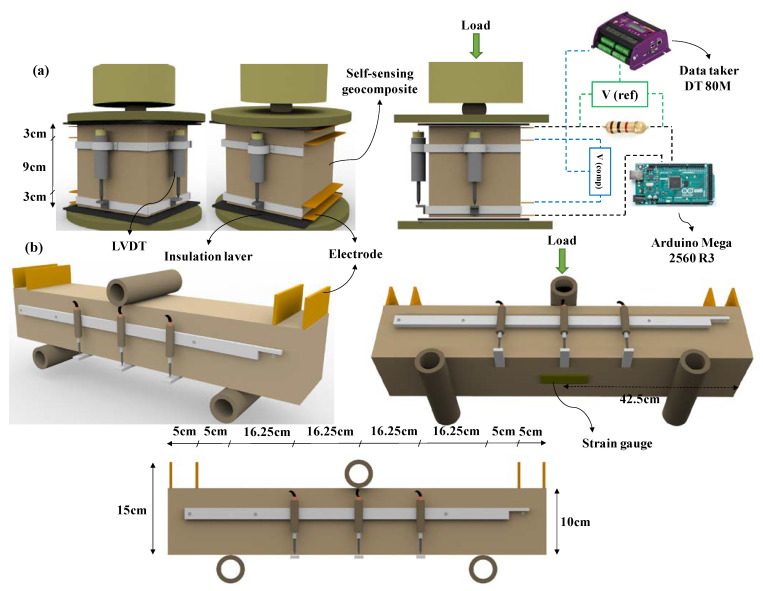
Test setup and electrode configuration: (**a**) compression, (**b**) flexural.

**Figure 6:**
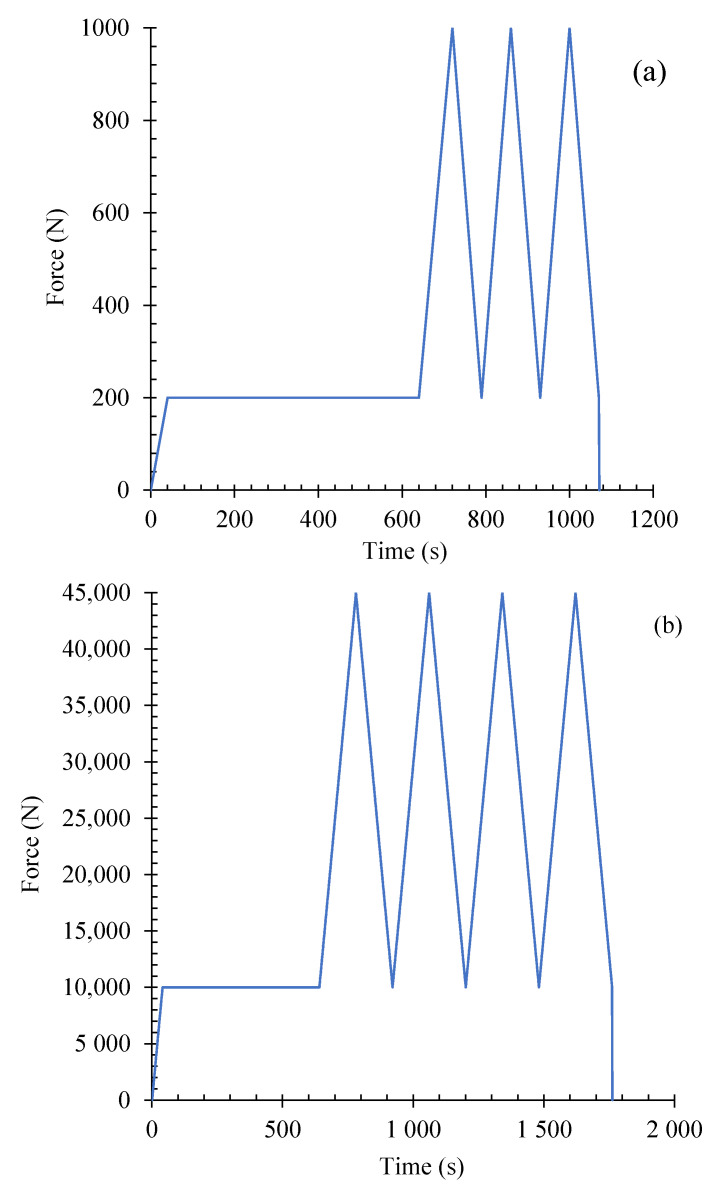
Cyclic loading patterns used for piezoresistivity behaviour investigations: (**a**) flexural mode, (**b**) compression mode.

The electrical percolation threshold for a large-scale beam with dimensions of 10 × 10 × 85 cm^3^ and a cubic 15 cm length specimen was calculated using the relations obtained by hybrid CNT/GNP. To investigate piezoresistivity, a four-probe method and direct current (DC) source was used (Figure 5) [3]. The four-probe method is highly recommended for piezoresistivity investigations in previous studies as a more accurate technique for eliminating the influential resistance from current lines, joints, interfaces and polarization effects [15,46,47,48]. The electrical properties and piezoresistivity behaviour of these specimens were evaluated under flexural (three points) and compression cyclic loading, as shown in Figure 6. A similar electrical circuit was used in both flexural and compressive loading modes, the details of which are the same as Section 2.3. As shown in Figure 5, the strain in compressive and flexural loading was measured by LVDT (RDP ACT 500A, accuracy: 10^−6^ m) and strain gauge (TML/PL 60, gauge resistance: 120 ± 3 Ω, accuracy: 10^−6^ m), respectively.

Equation (4) was used to obtain the fractional changes in electrical resistivity (*FCR*) for the following assessment of composite piezoresistivity:

(4) $$FCR=\frac{\rho \left(t\right)-\rho_{0}}{\rho_{0}}$$

where *ρ*_(*t*)_ is the resistivity during the test at time t and ρ_0_ is the initial electrical resistivity (before loading). The gauge factor (*GF*) is also defined as the relative change in electrical resistivity over the strain (Equation (5)) in order to evaluate the sensitivity of composites:

(5) $$GF=\frac{FCR}{\varepsilon }$$

where *ε* is the axial and flexural strain for the compressive (mean of three LVDT) and flexural (strain gauge) loading modes, respectively.

### 2.5. Mechanical Properties

The three-point flexural strength of the beam was also measured following EN 1015-11 [6]. Three cubic specimens (100 × 100 × 100 mm^3^) were also cut from the beam after the test, and their axial compression strength was evaluated along with the large cubic (150 × 150 × 150 mm^3^) specimen. Compressive and flexural elastic moduli were also calculated [6]. The results are obtained by the mean of 3 specimens.

## 3. Results

### 3.1. Effects of Length Changes

**Figure 7:**
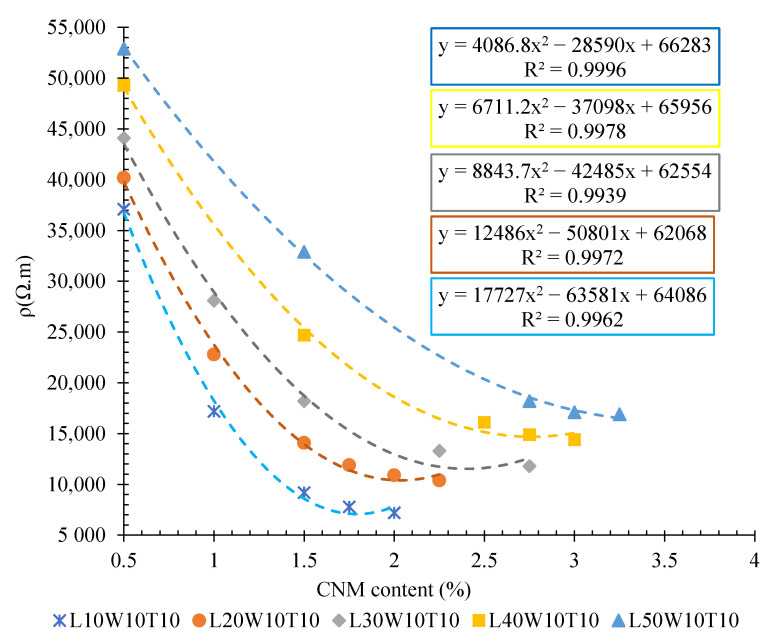
Volumetric electrical resistance of CNT/GNP-reinforced specimens with different lengths (surrounded between electrodes).

The volumetric electrical resistances of specimens with different lengths (surrounded between electrodes) are illustrated in Figure 7. The specimens were composed of different content of hybrid CNT/GNP.

**Figure 8:**
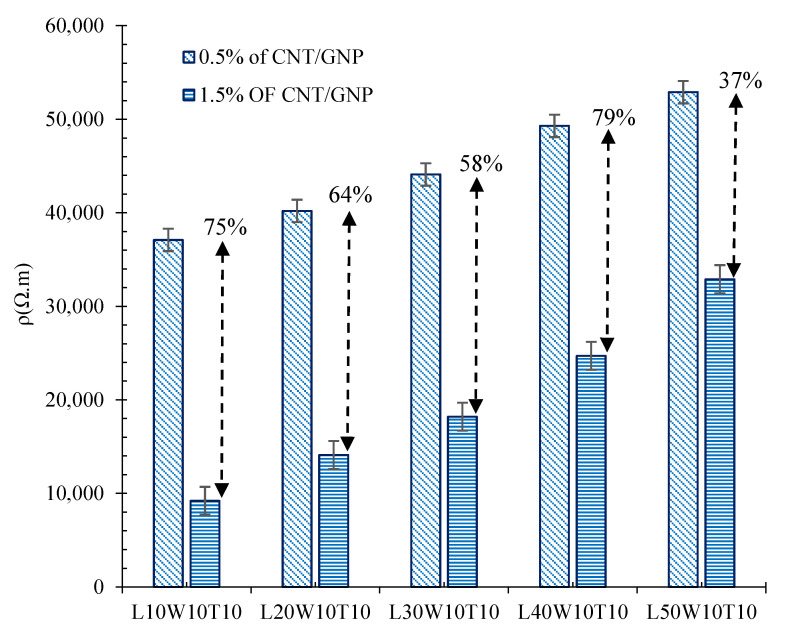
Volumetric electrical resistance of the specimens with different lengths and reinforced by 0.5 and 1.5% CNT/GNP.

As can be observed, increasing the length of the matrix (surrounded between electrodes) significantly increased the electrical resistivity of the cementitious composite. Indeed, increasing the length of the specimen, which was composed of 0.5% CNT/GNP, from 10 cm to 20, 30, 40, and 50 cm, caused an increase in volumetric electrical resistance of approximately 10, 19, 33, and 43%, respectively. Meanwhile, these amounts for the specimens containing 1.5% hybrid CNT/GNP were around 53, 97, 168, and 257%, respectively. In fact, the increase in the percolation threshold with the increase in the matrix length might have been one of the reasons for the significant increase in the resistivity of the specimens containing 1.5% CNMs compared to that of the samples containing 0.5% CNMs. To better interpret this issue, the results of electrical resistivity for the specimens with different lengths (surrounded between electrodes), containing 0.5% and 1.5% CNT/GNP, are presented in Figure 8. In the specimens with lower lengths, due to the lower percolation threshold (and its proximity to 1.5%), the degree of resistivity reduction caused by increasing the CNM content from 0.5% to 1.5% was higher than in longer specimens.

In fact, increasing the length of the specimen surrounded by two electrodes means increasing the distance between the electrodes. Accordingly, an increase in the electrical resistance of composites that is affected by increasing the distance between the electrodes has been reported in several studies [27,28]. Generally, the physical and geometrical parameters of the composite have significant effects on the electrical resistance of the cementitious composite. Obviously, by increasing the length of the specimen surrounded by two electrodes and consequently increasing the volume of the covered matrix, the electrical resistance will be increased. Although the results of the present study show an increase in percolation threshold with increasing electrical resistance, the lack of a clear numerical relationship between the percolation threshold and electrical resistance poses challenges against interpreting the percolation threshold changes and electrical resistance based on physical parameters. However, by a simple approach, it is conceivable that increasing the matrix volume will increase the required conductive filers to achieve a lower and approximately constant electrical resistance. This issue required further investigation.

As shown in Figure 7, the slope of the curves generally decreased with increasing length of the specimens (surrounded between electrodes), which might demonstrate the lower performance of these CNMs in terms of increasing the electrical conductivity of the large specimens. Hence, this could be evidence that there is a relationship between the dimensions of the conductive filler and the matrix, which could then be manipulated to achieve high conductivity. However, this issue needs to be further assessed.

**Figure 9:**
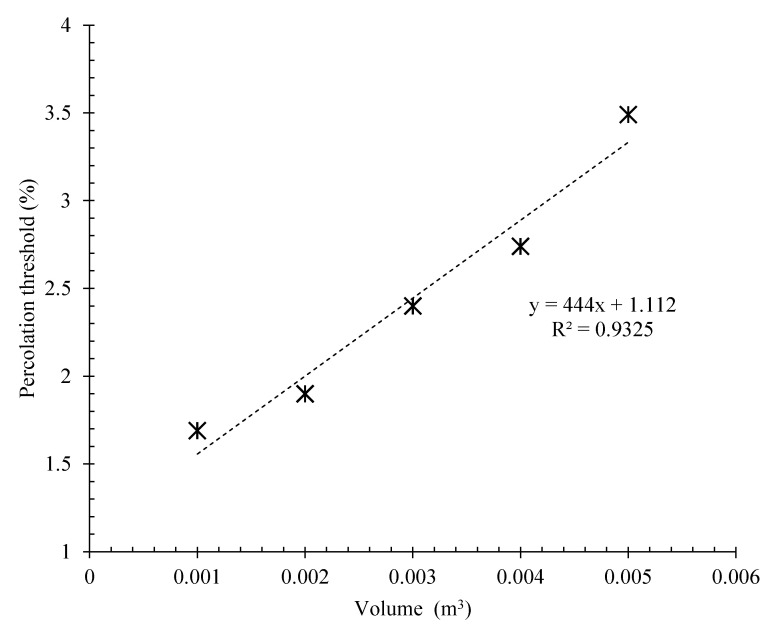
Relation between electrical percolation threshold and matrix volume of CNT/GNP reinforced specimens with different lengths.

The electrical percolation threshold of the specimens was also obtained from the derivative of the relationship between the percentage of CNMs and their electrical resistivity, as depicted in Figure 9. As can be observed, the percolation threshold of the composite increased with increasing length and, consequently, the volume of the matrix (surrounded between electrodes). A well-defined relationship between the percolation threshold and matrix volume was also obtained with a low approximation using a linear function.

### 3.2. Effects of Cross-Section Changes

**Figure 10:**
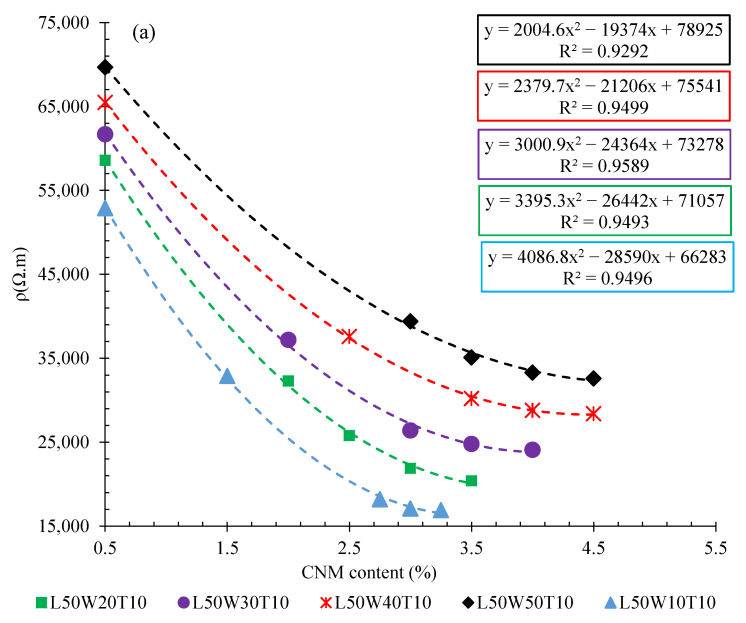

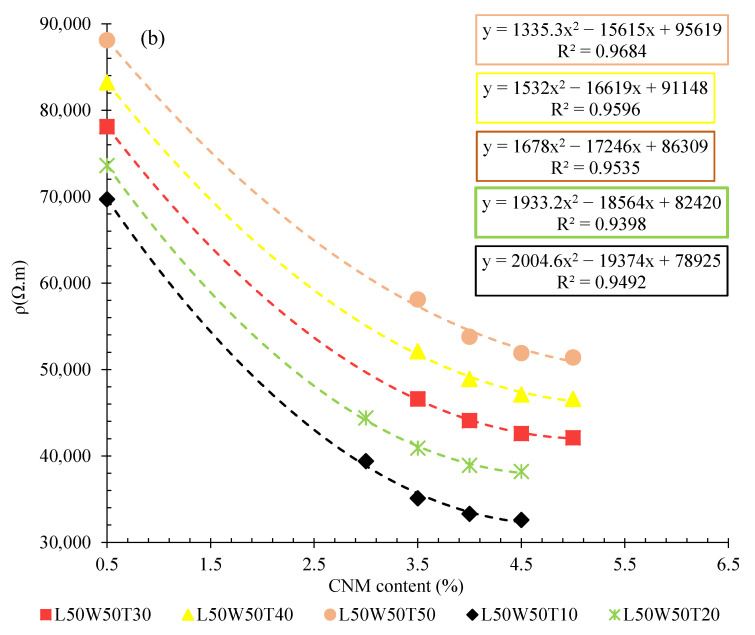
Volumetric electrical resistance of CNT/GNP reinforced specimens with: (**a**) different width, (**b**) different thickness.

The electrical resistivity of the CNT/GNP-reinforced specimens with different section widths and thicknesses are shown in Figure 10. It should be noted that widths and length of matrix cross section (thicknesses) reflect the surface area of the electrodes.

**Figure 11:**
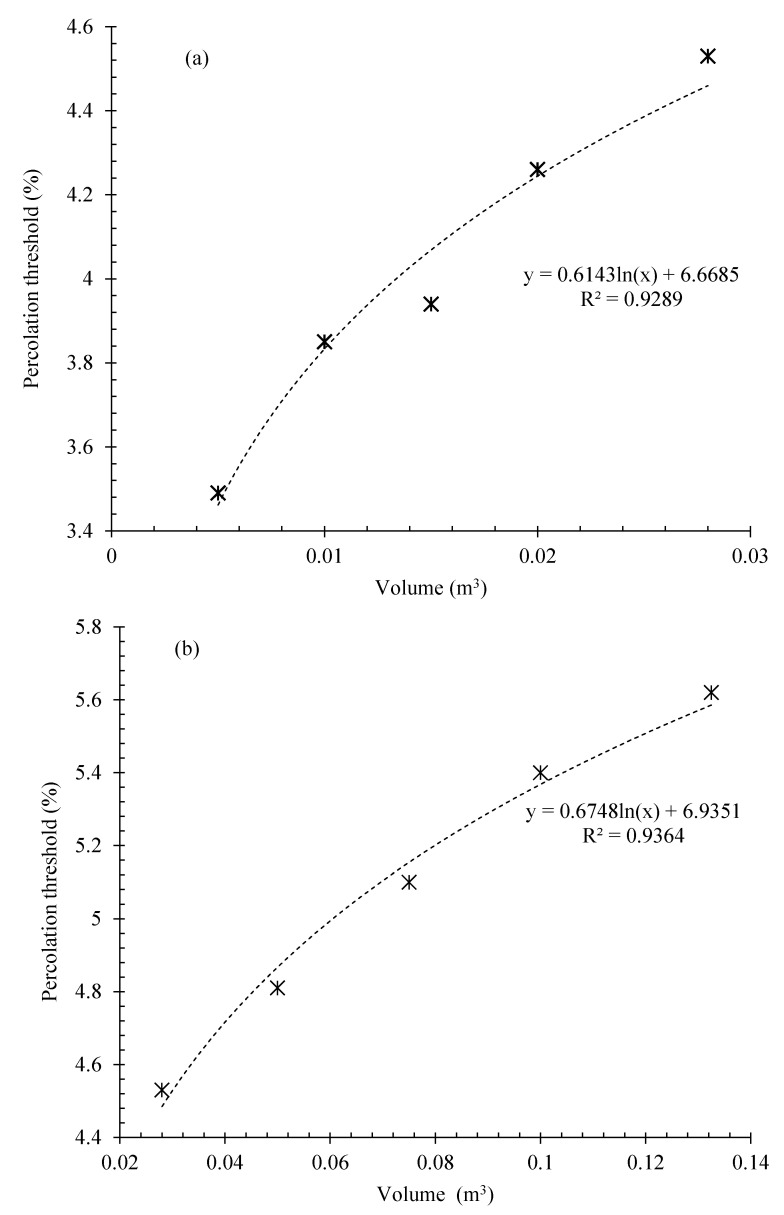
Relation between electrical percolation threshold and matrix volume for CNT/GNP reinforced specimens with: (**a**) different width, (**b**) different thickness.

With respect to changes in the cross-sectional area, increasing the width of the specimen containing 0.5% hybrid CNT/GNP from 10 cm to 20, 30, 40, and 50 cm increased the volumetric electrical resistance by approximately 10%, 16%, 23%, and 31%, respectively. The results also showed that by increasing the CNM content to 1.5%, the effect of changes in the width of the cross-section on the increase in electrical resistivity also increased, in such a way that increasing the specimen width to 20, 30, 40, and 50 cm led to an increase in the resistivity by approximately 19, 32, 49, and 62%, respectively. The results of the electrical resistance for specimens with different thicknesses also showed similar trends. As can be seen in Figure 11b, the volumetric electrical resistance of the specimens containing 0.5% CNMs with thicknesses of 20, 30, 40, and 50 cm increases by approximately 12%, 19%, 27%, and 34%, respectively, compared to the specimen with 10 cm thickness.

The calculated percolation thresholds for the specimens with different cross-sectional widths and thicknesses are shown in Figure 11a,b. The relationship between the electrical percolation threshold and variation in the cross-sectional area of the matrix was also expressed by the logarithmic and linear functions.

As can be observed, increasing the width or thickness of the matrix cross-section increased the percolation threshold of the composite. However, the slope of the curve of the thickness variation was lower than the width changes, which shows that the width changes had a greater effect on the electrical percolation threshold.

**Figure 12:**
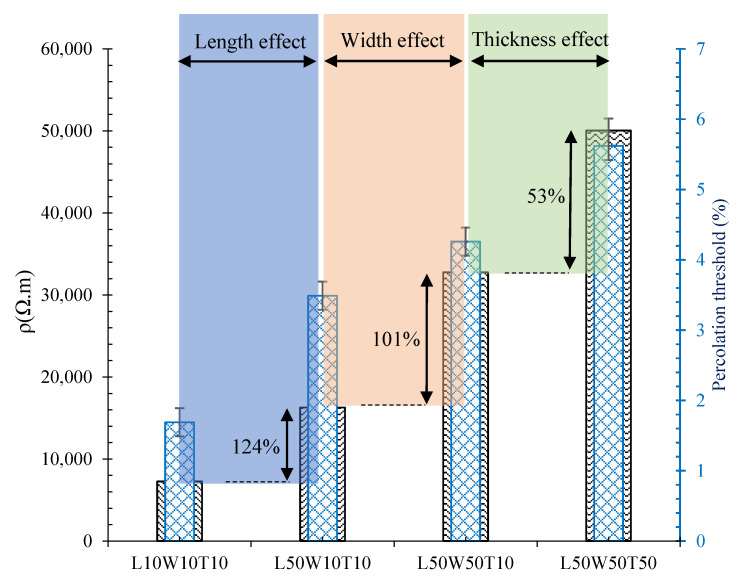
Electrical resistivity of specimens reinforced with hybrid CNT/GNP at the percolation threshold content.

In order to better evaluate the contribution of each dimension change in the variation of electrical resistivity, and consequently the percolation threshold, the electrical resistivity of the 10 × 10 × 10 cm^3^, 50 × 10 × 10 cm^3^, 50 × 50 × 10 cm^3^, and 50 × 50 × 50 cm^3^ specimens were calculated, as shown in Figure 12.

Clearly, the effect of length changes was greater than the width and thickness changes in such a way that increasing the length, width, and thickness of the specimens from 10 cm to 50 cm caused an increase in electrical resistivity of approximately 124, 101, and 53%, respectively.

### 3.3. Effects of Volume Changes

**Figure 13:**
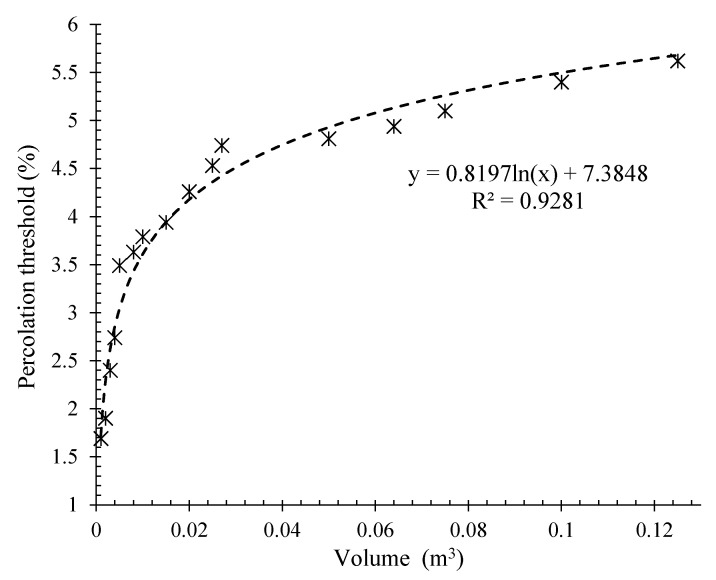
Relation between electrical percolation threshold and matrix volume for CNT/GNP reinforced specimens with different volumes.

Figure 13 shows the variation in the electrical percolation threshold of the specimens reinforced with CNT/GNP versus the matrix volume (surrounded between electrodes) changes by considering specimens with dimensions of 20 × 20 × 20 cm^3^, 30 × 30 × 30 cm^3^, and 40 × 40 × 40 cm^3^.

Based on Figure 13, there is a well-defined trend between the matrix volume (surrounded between electrodes) and its electrical percolation threshold, which is expressed by a logarithmic function. With this proposal, design engineers can easily predict the electrical percolation threshold, which is one of the key factors in smart composite design and development [49,50].

### 3.4. Effects of Fillers’ Aspect Ratio

**Figure 14:**
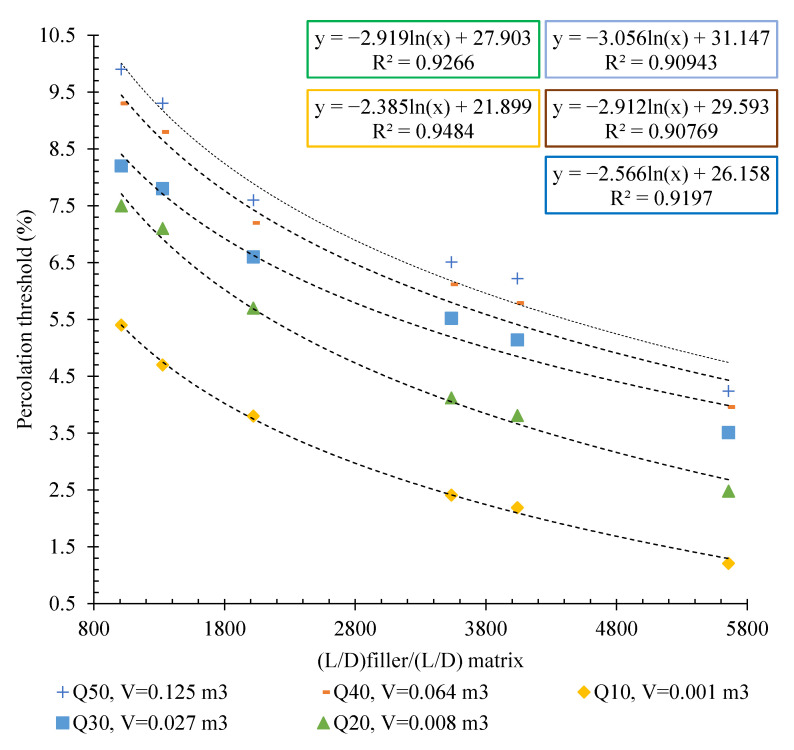
The relative aspect ratio of the carbon-based fillers vs. percolation threshold for the specimens with different volumes.

As mentioned, in order to evaluate the effect of the filler aspect ratio on the percolation threshold of the matrix, different content of the three different types of CFs and CNTs, which varied in terms of their length, were used as conductive fillers. Square cubic specimens with different volumes were also used to remove the effects of the matrix aspect ratio. The variation of percolation threshold relative to the aspect ratio of the fillers for the specimens are illustrated in Figure 14.

As can be observed, increasing the aspect ratio of the conductive fillers relative to the matrix caused a reduction in the electrical percolation threshold percentages. The same trend was reported in the literature [45,51,52]. They reported that the percolation threshold value increases when the aspect ratio decreased. Chiarello and Zinno [51] expressed that the threshold value is defined as the conductive filler volume where the clusters of the conductive filler start to be in contact with one another, forming the conductive network through the insulating matrix.

In fact, to form the conductive network, a greater content of short filler than long filler is necessary, because the latter are more likely to connect themselves to form the percolation path.

Another point of interest is that by evaluating the slope of the graphs for different matrix volumes, it can be seen that increasing the aspect ratio of the filler relative to the matrix had a greater effect on reducing the percolation threshold in larger specimens. In other words, at larger volumes, fillers with higher aspect ratios exhibited higher performance.

**Figure 15:**
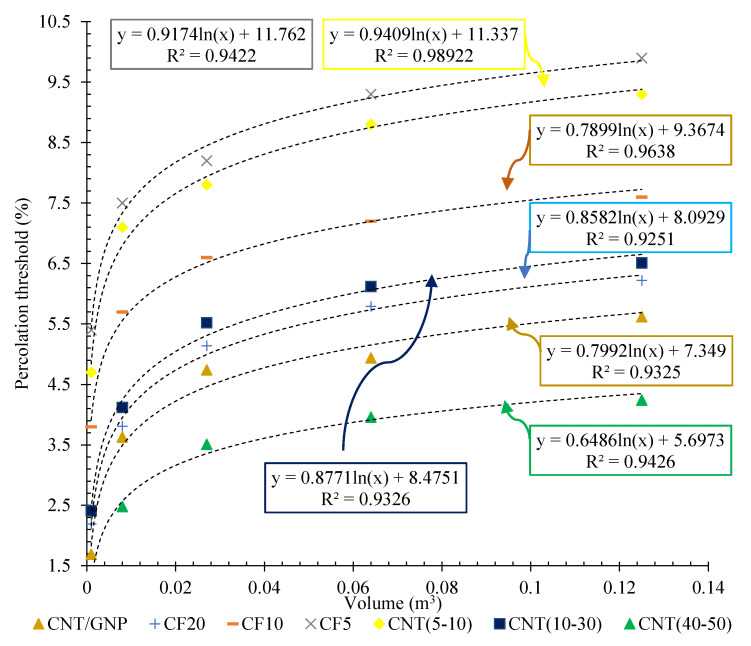
The percolation threshold vs. matrix volume (surrounded between electrodes) for the different CF and CNT reinforced specimens.

The results of the percolation threshold for the specimens reinforced with CFs and CNTs of different lengths are also shown in Figure 15.

The results clearly showed that increasing the matrix volume (surrounded between electrodes) of all types of conductive fillers consistently increased the electrical percolation threshold percentage. As can be observed, in an identical matrix volume, the lowest percolation threshold was obtained for the specimen composed of CNT (40–50). In fact, the prioritisation of conductive fillers in terms of their performance in reducing the electrical percolation threshold of cementitious geocomposites, from best to worst performance, was CNT (40–50), CNT/GNP, CF (20), CNT (10–30), CF (10), CNT (5–10), and CF (5).

Al-Dahawi et al. [22] also assessed the effects of the aspect ratio of conductive filers on the percolation threshold of a cementitious mortar. They incorporate the different content of MWCNT (20–30 nm of diameter and 10–30 µm of length), CF (7.5 µm of diameter and 12 mm of length), GNP (5 µm of diameter and 5–100 nm of thickness), and carbon black particles (CB, 20–100 nm of diameter) into a 15 cm length of the cubic specimens with a water-to-cement ratio of 0.27. They measure the electrical resistivity of the specimens with two probes method and using a concrete resistivity meter with uniaxial configuration, which employs the alternating current (AC) technique. They reported the lower percolation threshold of 0.55%, 1.00%, 2.00%, and 2.00% for the CNT, CF, GNP, and CB, respectively.

### 3.5. Piezoresistivity Investigations

#### 3.5.1. Flexural Loading

**Figure 16:**
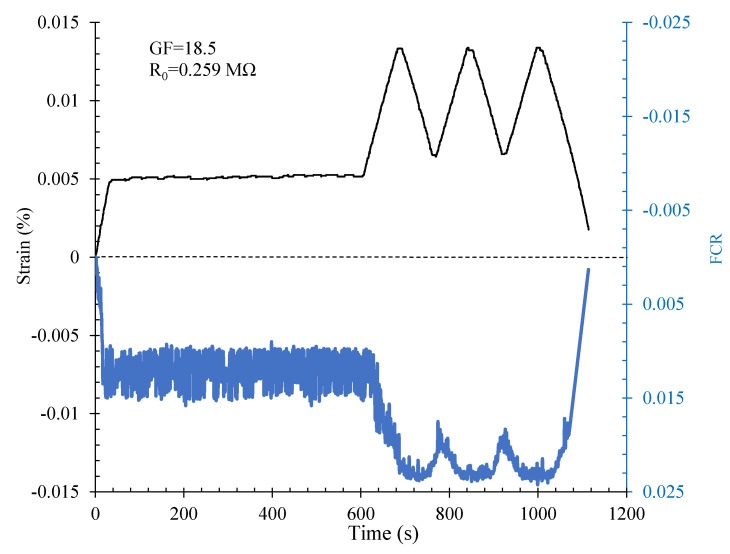
Piezoresistivity response of the beam under flexural cyclic loading.

The percolation threshold of a 10 × 10 × 85 cm^3^ beam was calculated using the obtained relation (Figure 13). The percolation threshold for this specimen was approximately 3.47% for the hybrid CNT/GNP. The piezoresistivity behaviour of this specimen under flexural cyclic loading is illustrated in Figure 16. The initial electrical resistivity of this specimen was approximately 3237 Ω.m. A proper correlation was obtained between the strain changes and the fractional change in electrical resistivity (FCR) which indicated the high potential of the SCG in terms of strain and/or stress detection. As shown in Figure 16, the FCR consistently increases with increasing load and decreases with decreasing load. Indeed, increasing the load and, consequently, the strain, caused the destruction of the conductive paths formed by the conductive fillers (CNT/GNP) which led to an increase in the electrical resistivity of the composite. A similar trend was reported in the literature [6].

The gauge factor (GF) for this specimen was approximately 18.5, which demonstrates the excellent sensitivity of this composite to strain and stress. However, due to the noise of the signal, the mean of the FCR values was used to calculate the sensitivity.

#### 3.5.2. Compression Loading

**Figure 17:**
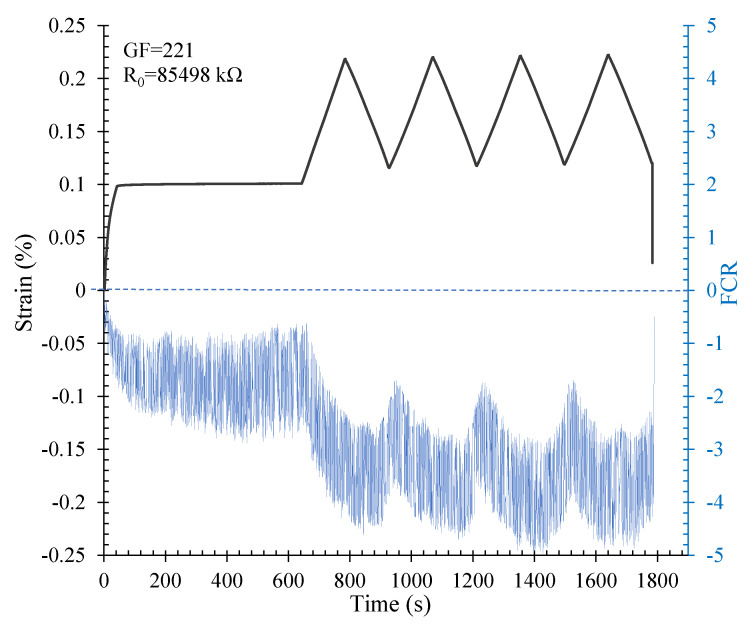
Piezoresistivity response of the cubic specimen under compression cyclic loading.

In the compression loading mode, a cubic specimen with dimensions of 15 cm was used. The percolation threshold for this volume was approximately 2.7%. The piezoresistivity responses of this specimen under compression cyclic loading are shown in Figure 17.

The electrical resistivity of this specimen was approximately 12,750 Ω.m. In the compression loading mode, the results showed a proper correlation between the variation in FCR and strain. Negative values were measured for the FCR because of a decrease in the electrical resistance during compression loading relative to the initial electrical resistance of the composite [40]. In fact, the paths formed by conductive fillers got closer together during compression loading, which resulted in the decreased electrical resistivity and consequently enhanced the electrical conductivity of the composite. This specimen also indicated a high level of sensitivity to the compression loading in such a way that a gauge factor of approximately 221 was also obtained for this SCG.

### 3.6. Mechanical Investigations

#### 3.6.1. Mechanical Properties of the Beam

**Figure 18:**
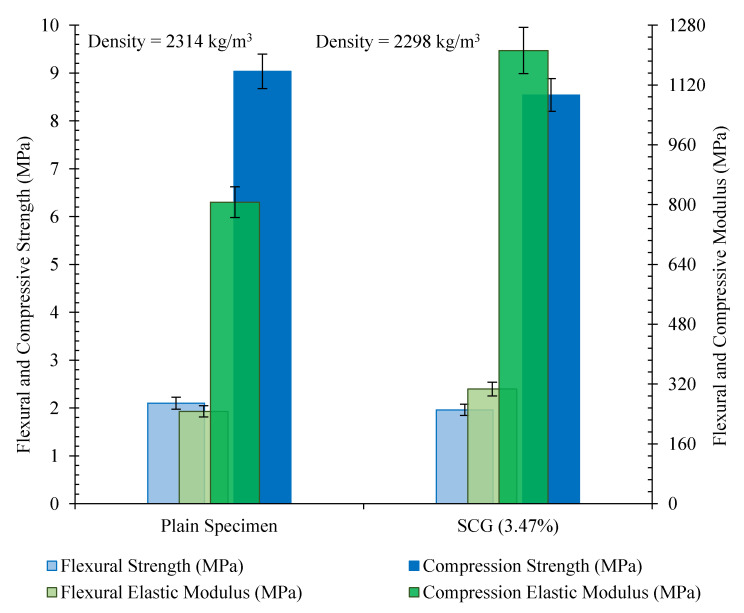
Mechanical properties of the 10 × 10 × 85 cm^3^ beam reinforced with 3.47% of CNT/GNP (SCG 3.47%).

The mechanical properties of the 10 × 10 × 85 cm^3^ beam reinforced with 3.47% CNT/GNP (SCG 3.47%) are illustrated in Figure 18. The results did not show significant changes in the flexural and compression strengths of the specimen (SCG 3.47%) when compared to the plain specimen. However, reinforcing the plain specimen with 3.47% CNT/GNP caused an increase in the flexural elasticity modulus of approximately 25%. Furthermore, the incorporation of 3.47% hybrid CNT/GNP into the plain specimen increased the compression elasticity modulus by approximately 50%. A similar trend in terms of the ability of CNT/GNPs to increase the moduli has been reported in previous studies [3,6].

#### 3.6.2. Mechanical Properties of the Cubic Specimen

**Figure 19:**
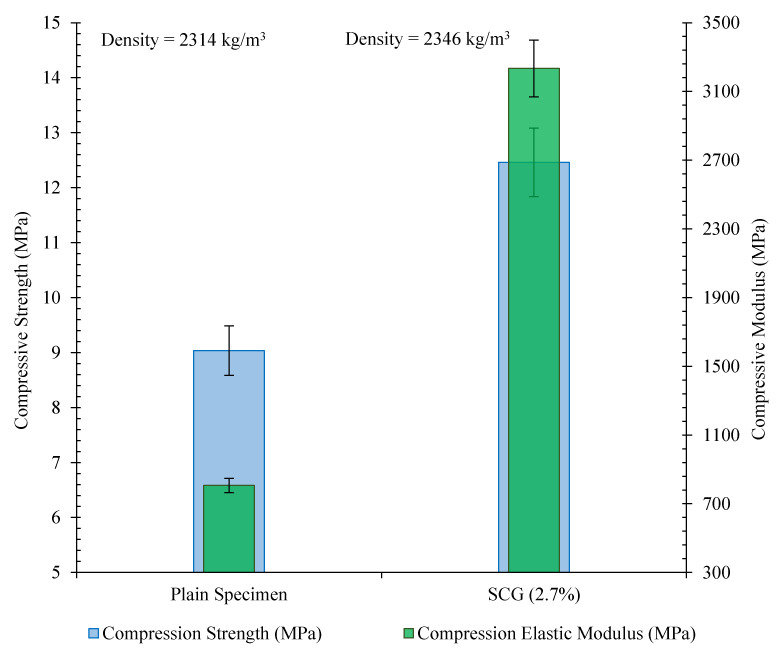
Mechanical properties of the 15 cm cubic specimen reinforced with 2.4% of CNT/GNP (SCG 2.7%).

The compression strength of the 15 cm cubic specimen composed of 2.4% CNT/GNP (SCG 2.7%) is shown in Figure 19. The compressive strength of the specimen reinforced with 2.4% CNT/GNP increased by approximately 38% compared to that of the plain specimen. The compression elasticity modulus of the specimen (SCG 2.7%) obtained was also three times larger than that of the plain specimen.

Generally, reinforcing cementitious composites with CNTs and GNPs improved the physical and mechanical properties of the hardened composite through its filler function and bridging and/or through deviation of the cracks [3,6,29,30,34,53]. A hybrid combination of one dimensional CNT and two dimensional GNP with different geometrical shapes and sizes can fill a wider range of pores and spaces between the cement particles. Then, by starting the cement hydration process, the nanoparticles buried among the hydration products can improve the physical properties and reinforce the microstructure of the composite [54]. The CNTs bridge across the cracks and the GNPs effectively divert them, thereby reducing the accumulation of cracks. These phenomena greatly increase the load-carrying capacity and forces needed for the development of crack and element failures. When a crack meets a buried CNT or GNP, it is blocked, and a new load increase is needed for further propagation. In addition, CNTs and GNPs could act as nucleation agents and increase the hydration rate in cementitious composites [55]. However, an excessive increase in CNM content caused agglomeration and consequently reduced the physical and mechanical performances of the composite [30].

## 4. Conclusions

This was an extensive study designed to clarify the co-effects of electrode space and surface area as well as filler scales on the electrical percolation threshold, which is one of the most important parameters involved in the design of self-sensing cementitious composites for the field applications. In this route, the electrical conductivity of several specimens of different shapes, sizes, and content of carbon conductive fillers such as CNT, GNP, and CF, were measured. The electrodes were installed on both ends of specimens to completely surround the matrix. In addition, conductive fillers with different aspect ratios were used to investigate the effects of filler scale on the electrical percolation threshold. In this study, stabilised sand with 10% cement was used, and the specimens were fabricated using the compaction method. Furthermore, the numerical relations between the electrical percolation threshold and matrix dimensions were expressed for different conductive fillers.

Finally, the electrical percolation threshold of two large-scale specimens with different shapes (a 10 × 10 × 85 cm^3^ beam and a 15 cm cube) were predicted through the obtained numerical relations, and their piezoresistivity performances were investigated under flexural and compression cyclic loading. In addition, the mechanical properties of these specimens were evaluated and the following conclusions were drawn:

- The volumetric electrical resistance of conductive specimens with different dimensions showed that the changes in the length, width, and thickness of the matrix surrounded between electrodes had a significant effect on the changes in the electrical percolation threshold of the composite. Generally, increasing the volume of the surrounded matrix caused an increase in the percolation threshold.
- The investigations showed that the effects of length changes in the surrounded matrix in the percolation threshold were greater than the effects of its width and thickness changes, in such a way that increasing the length, width, and thickness of the surrounded matrix from 10 cm to 50 cm caused an increase in electrical resistivity by approximately 124%, 101%, and 53%, respectively.
- Generally, increasing the aspect ratio of the conductive fillers causes a reduction in the electrical percolation threshold of the cementitious composite.
- In this study, the prioritisation of conductive fillers in terms of their performance in reducing the electrical percolation threshold of cementitious geocomposites from most to least effective was as follows: CNT (40–50), CNT/GNP, CF (20), CNT (10–30), CF (10), CNT (5–10), and CF (5).
- The 10 × 10 × 85 cm^3^ beam and 15 cm cubic specimens with hybrid CNT/GNP content of 3.47 and 2.74%, respectively (obtained by the numerical relation presented in this study), showed a proper piezoresistive response under flexural and compression loading, respectively.
- Incorporation of 3.47% CNT/GNP into the specimens (beam) did not cause a considerable change in the flexural and compression strengths of the specimen. However, it increased the flexural and compression elasticity modules by approximately 25% and 50%, respectively.
- Reinforcing the cubic specimen with 2.4% CNT/GNP improved the compression strength by approximately 38%. The compressive elasticity modulus of this specimen was three orders of magnitude greater than the plain composite.

In summary, we believe that this novel approach could contribute to the new era of self-sensing geocomposite design for intelligent structures. Indeed, the results of the present study illuminate the path for passing from small-scale laboratory investigations towards large-scale self-sensing geocomposites.

## Figures and Tables

**Table 1:** Characterization of multi-wall carbon nanotube (MWCNT), graphene nanoplatelets (GNP), and short carbon fibre (CF).

Characterization	Carbon Content (%)	Length (µm)	Outside Diameter (nm)	Density (g/cm^3^)	Layers	Form
CNT (5–10)	>98	5–10	7–15	2.21	-	Black powder
CNT (10–30)	>98	10–30	<8	2.27	-	Black powder
CNT (40–50)	>95	40–50	8–15	2.34	-	Black powder
GNP	>99.5	5–10	4–20	2.25	10 < n < 60	Grey powder
CF (5)	>93	5000	7000	1.87	-	Black microfibre
CF (10)		10,000				
CF (20)		20,000				

**Table 2:** Sand particle size distribution.

**Mesh size (mm)**	0.08	0.16	0.5	1	1.6	2
**Cumulative retained (%)**	99 ± 1	87 ± 5	67 ± 5	33 ± 5	7 ± 1	0
**Specific gravity G_s_**	2.67		**Cu ^a^**	7.5	**Cc ^b^**	1.8

^a^ Uniformity coefficient, ^b^ Curvature coefficient.

## Data Availability

Some or all data, models, or code that support the findings of this study are available from the corresponding author upon reasonable request.
